# Can chimpanzees conceive of mutually exclusive future possibilities? A Comment on: ‘Chimpanzees prepare for alternative possible outcomes’ (2023), by Engelmann *et al.*

**DOI:** 10.1098/rsbl.2023.0409

**Published:** 2024-06-26

**Authors:** Jonathan Redshaw, Thomas Suddendorf

**Affiliations:** ^1^ School of Psychology, The University of Queensland, Saint Lucia, Queensland 4067, Australia

**Keywords:** possibilities, reasoning, logic, modal cognition, prospection, cognitive evolution

*Biol. Lett.* **19**: 20230179 (Published online 21st June 2024). (https://doi.org/10.1098/rsbl.2023.0179)

## 1. Introduction

The ability to think about future events and consider mutually exclusive possibilities is a bedrock of human cognition—essential for adaptive contingency planning, logical analysis and moral reasoning—and the nature, development and evolution of this ability have recently attracted considerable attention [1–4]. In their *Biology Letters* article, Engelmann *et al*. [5] report evidence that chimpanzees also prepare for multiple possible outcomes and suggest that they too represent exclusive-OR relations. Here, we point out critical concerns with their study and caution against such a rich interpretation of their findings: leaner explanations are available.

## 2. Concerns with the study rationale

Engelmann *et al*.’s study used an adaptation of our forked tube task [6], in which an experimenter drops a reward into an inverted Y-shaped tube with two exits, such that a participant must simultaneously cover both exits with one hand each to guarantee successfully catching it (also [7]). Across our studies, we have found little evidence that non-human primates insightfully prepare for mutually exclusive possibilities in this simple context [6,8,9]. Englemann *et al*., however, criticize our methodology on the basis that covering both exits ‘does not come naturally to chimpanzees’.

Although we agree it is possible that covering two exits in our tube tasks is physically difficult for non-human primates (even if they must naturally coordinate their hands as they climb trees), we do not think such a difficulty can fully explain the existing pattern of results. In our experiments with 14 primates (five chimpanzees across two studies [6,8], five orangutans in one study [6], and four monkeys in one study [9]), we found that three chimpanzees, one orangutan and one capuchin monkey spontaneously covered both exits despite never seeing that behaviour before. The target response is therefore clearly within the behavioural repertoire of primates, and our subjects did not require training or demonstration to evince it. Crucially, however, each of these five individuals regressed to covering one exit on subsequent trials, suggesting they had not attained the critical conceptual insight that covering two exits can guarantee success.

In critiquing our task, Engelmann *et al*. [5] cite an independent follow-up study, which argued that covering two exits is generally challenging for chimpanzees [10]. Nonetheless, this study found that when two rewards were simultaneously dropped into two parallel tubes, chimpanzees spontaneously covered two exits on 24.4% of trials, seven times more often than when only one reward was unpredictably dropped into one of the two mutually exclusive tubes (on 3.4% of trials; see table 1 in [10]). Furthermore, the three individuals who learned to consistently cover both exits towards the end of the simultaneous trials (i.e. passing 90–100% of the final 10 trials) subsequently passed only 17–58% of mutually exclusive trials in a supplementary experiment. These patterns were observed even though the apes had to cover both exits to guarantee a reward in the mutually exclusive trials, but could simply cover one exit to guarantee a reward in the simultaneous trials (see box 1 in [11]). On the whole, although this study was framed as a challenge to the validity of our task, the data indicate that the primates’ trouble with preparing for mutually exclusive possibilities was at least partly in their minds and not simply in their hands.

## 3. Concerns with the results

Because of their assumption that chimpanzees may have difficulty covering two exits with one hand each, Engelmann *et al*.’s study involved a lengthy familiarization procedure to ensure that subjects had the basic capacity to perform a similar target behaviour: holding two platforms simultaneously. This target behaviour, despite the familiarization, was too difficult for some chimpanzees, and so three individuals had to be excluded (see supplementary material [5]). The preregistration also states that the remaining chimpanzees were given up to three attempts per trial if they failed to balance either platform and that trials were excluded if they did not (but the article did not report how often and in what conditions this actually occurred). Overall, while it would be difficult to make the case that Engelmann *et al*.’s target behaviour is any more natural to chimpanzees than covering two tube exits, we agree that, for the retained animals and trials, at least, their results cannot be explained by physical difficulties with the target behaviour.

Following familiarization, Engelmann *et al*. presented the chimpanzees with two baited platforms and either a single tube above one of the platforms or a forked tube with exits above both platforms. The chimpanzees could balance each platform to stop a dropped stone from tipping the bait off after exiting the tube. The assumption is that a rational agent understanding mutually exclusive possibilities would stabilize the platforms that were threatened by the dropping stone: the single platform over which there was the single tube in the control condition and both platforms in the test condition. The chimpanzees, however, did not just balance two platforms in the test condition. On their first trial, 7/16 chimpanzees balanced both platforms in the control condition and 8/16 did so in the test condition. Overall, they balanced both platforms on about half of the trials (49%) in the control condition, even though there was no rational reason to do so. This pattern demonstrates that stabilizing both platforms does not need to reflect a rational analysis of possibilities.

The authors’ rich interpretation hence rests only on the finding that chimpanzees in the test condition stopped both platforms from tilting on 72% of all trials, which was 23% more often than in the control condition. We agree it is possible that this difference was the result of chimpanzees foreseeing and understanding alternative possibilities, but this is not the only explanation available.

## 4. Concerns with the interpretation

We are not convinced that the paradigm could have even in principle provided strong grounds to suppose that chimpanzees can reason about mutually exclusive possibilities. This is because, rather than conceiving of the problem as involving an exclusive-OR relation (*the stone might remove the left reward OR the right reward*), the apes need only have approached the problem as if it involved a straightforward AND relation (*I want the left reward AND the right reward*). From this latter stance, the apes’ differential performance across conditions may have simply been driven by their increased attention to the two rewards when there was a tube exit over each. Recall that in the test condition, there were two prominent tube components pointing to two reward platforms, whereas in the control condition, there was only one prominent tube component pointing to one reward platform.

In other experiments, even where chimpanzees must consistently choose between one of two rewards (A or B), they sometimes point to both options (A and B), as reported in various studies including from one of the authors of Engelmann *et al*. [12–14]. Thus, simultaneously acting towards two rewards need not necessarily reflect an understanding of the logical relation that links them.

In contrast to Engelmann *et al*.’s task [5], our original tube tasks [6,8] involved only a *single* reward held above the tube apparatus during the preparation phase. There is thus no reason to suspect the primates could have approached our tasks as if they involved a straightforward AND relation. While we have found little evidence that non-human primates and 2-year-old human children can consistently prepare for two mutually exclusive possibilities on these tasks, we have found that children typically become able to do so between 3 and 5 years of age [6,8,15]. There may of course be other simple routes (unrelated to AND relations) by which a proportion of children pass this task, for instance by matching each hand with each exit without evaluating the possibilities entailed [16], or by representing the left and right exits as two independent possibilities (*maybe left, maybe right*) without representing the exclusive-OR relation that links them [17]. As we explicitly acknowledged in our original report, we are open to ‘leaner accounts of the older children’s performance’ [6]. Reassuringly, however, it is around 3–5 years that children also demonstrate increasing competence on other tasks designed to measure understanding of exclusive-OR relations [7,16–21] (although it remains to be seen whether children’s performances on these tasks are correlated).

Unlike the clear pattern of increasing success in preschool-aged children, studies have not consistently found that non-human primates pass tasks aimed at measuring understanding of exclusive-OR relations. Indeed, another recent report from Engelmann *et al*. [22]—which sampled from the same population of chimpanzees as in their *Biology Letters* study—found no compelling evidence of such understanding in a logical reasoning task that children convincingly pass by 5 years [19]. Notably, it has been proposed that the mixed evidence from primates [5,6,8–10,22–24] (and very young children [25–29]) can be explained by the notion that some tasks are more readily solved without understanding exclusive-OR relations than others [2,11,16,30].

## 5. Summary and future directions

We have raised concerns about Engelmann *et al*.’s [5] article on three grounds: (i) the rationale implies without sufficient evidence that chimpanzees’ poor performance on similar previous tasks can be entirely explained by physical difficulties, (ii) the chimpanzees’ high rate of balancing two platforms in the control condition shows that this behaviour need not reflect a rational analysis of possibilities, and (iii) the chimpanzees might have approached the task as if it involved a straightforward AND relation between two rewards rather than an exclusive-OR relation between two possibilities, with the relatively greater performance in the experimental condition driven by increased attention to the two rewards when there was a tube exit above each. And although our own positive evidence from human children [6,8,15] remains open to other explanations that do not entail an understanding of exclusive-OR relations [16,17], it is clear that children do acquire this understanding at some point during development—with many studies (including our own) pointing to a critical transition period between 3 and 5 years.

Moving forward, the study of non-human animals’ capacities to represent possibilities will benefit from a nuanced engagement with existing interpretations and, fittingly, an openness to alternative resolutions. Although some of the authors of Engelmann *et al*. have elsewhere suggested ‘Redshaw and Suddendorf claim that chimpanzees are not able to represent alternative possibilities’ [23], we did not in fact make such a categorical claim, and were careful to acknowledge that there may be other reasons why great apes failed our task [6]. We later proposed that non-human animals may in fact *represent alternative possibilities* without necessarily representing the exclusive-OR relation that links such possibilities [11] (a proposal that was subsequently bolstered by evidence suggesting that the rodent hippocampus encodes dual possibilities via distinct elements of the theta rhythm [31]). But, regardless of the veracity of this particular interpretation, it is essential that all possible interpretations remain on the table until compelling evidence rules them out.

## Data Availability

This article has no additional data.
